# Push It to the Limit: Identification of Novel Amino Acid Changes on the Acetolactate Synthase Enzyme of Rice That Putatively Confer High Level of Tolerance to Different Imidazolinones

**DOI:** 10.3389/fbioe.2020.00073

**Published:** 2020-02-14

**Authors:** Giseli Buffon, Thainá Inês Lamb, Mara Cristina Barbosa Lopes, Raul Antonio Sperotto, Luís Fernando Saraiva Macedo Timmers

**Affiliations:** ^1^Graduate Program in Biotechnology, University of Taquari Valley – Univates, Lajeado, Brazil; ^2^Biological Sciences and Health Center, University of Taquari Valley – Univates, Lajeado, Brazil; ^3^Rice Research Institute, Cachoeirinha, Brazil

**Keywords:** alanine-scanning, herbicide, molecular docking, point mutation, bioinformactics

## Abstract

Advancements in genetically modified herbicide tolerance technology opened a new way to manage weed populations in crop fields. Since then, many important genetically modified crops that are tolerant to various herbicides have been developed and commercialized. Herbicides primarily act by disrupting key enzymes involved in essential metabolic or physiological processes associated with growth and development of plants. Most of the herbicide tolerant plants have been developed by introducing point mutations (non-GM approach) in the target site of herbicide action, due to the advantage of easier registration/release for commercial cultivation as well as wider public acceptance. Of the various herbicides, Imidazolinones are probably the most widely targeted ones for developing herbicide tolerant crops through non-GM approach. In rice, different mutant lines presenting amino acids changes in acetolactate synthase (ALS) have the ability to tolerate different Imidazolinones, including point mutations of Glycine to Glutamate in position 628, Serine to Asparagine in position 627, and a double mutation Tryptophan to Leucine in position 548/Serine to Isoleucine in position 627. The use of specific herbicides in combination of these mutant lines provides a reliable approach to eliminate weeds in the fields. However, the continuous overuse of a single herbicide multiple times in a growing season increases the potential risk of evolution of resistant weeds, which has become a major concern in agriculture worldwide. For this reason, the development of novel mutations in ALS (Os02g30630) to generate rice plants more tolerant to Imidazolinones than the available mutant rice lines is still a hot topic in plant-herbicide interaction field. Keeping that in mind, we carried out molecular docking experiments of Imidazolinone herbicides imazapic, imazapyr, imazaquin, and imazethapyr to evaluate the interaction of these molecules in the binding cavity of ALS from rice, being able to identify the most important amino acids responsible for the stability of these four herbicides. After introducing point mutations in these specific positions (one at a time) using Alanine scanning mutagenesis method and recalculating the effect in the affinity of herbicide-ALS interaction, we were able to propose novel amino acid residues (mainly Lysine in position 230 and Arginine in position 351) on the structure of ALS presenting a highest impact in the binding of Imidazolinones to ALS when compared to the already known amino acid mutations. This rational approach allows the researcher/farmer to choose the number of point mutations to be inserted in a rice cultivar, which will be dependent on the type of Imidazolinone used. To obtain a rice cultivar capable to tolerate the four Imidazolinone tested at the same time, we suggest six amino acid mutations at positions Val170, Phe180, Lys230, Arg351, Trp548, and Ser627 in the OsALS1.

## Introduction

Rice is considered an essential sources for global food security, being the staple food for approximately 50% of the world’s population (Muthayya et al., 2014; FAO, 2017). One of the most popularized seeding method (the direct one) saves labor and time, but leads to the production of weedy plants in paddy fields (Piao et al., 2018). According to Fartyal et al. (2018), one of the most impactful problem to rice culture is the presence of weeds, since they compete for nutrients, light, and other important resources. Furthermore, a critical impact on crop yield, plant’s survival and productivity can be observed due to infestation of weeds. Among available strategies, chemical herbicides are normally the first choice, since they are least expensive and most effective than other options (Nandula et al., 2005; Mithila and Godar, 2013). Therefore, herbicides are important constituents of modern integrated weed management system (Fartyal et al., 2018), as well as herbicide-resistant crops have had a profound impact on weed management (Duke, 2015; Maroli et al., 2016). The tolerance of crops to herbicide have been described by three mainly mechanisms, that are (i) “tolerance at the site of action,” (ii) “metabolic detoxification,” and (iii) “prevention of the herbicide from reaching the action site” (Tan et al., 2005). Therefore, to develop crops with herbicide tolerance is important to keep in mid one or more of these three mechanisms.

With the advance in herbicide tolerance technology, a new approach to manage weed populations in crop fields have been implemented. Since then, many important genetically modified crops tolerant to various herbicides have been developed and commercialized (Green and Owen, 2011; Fartyal et al., 2018). The mechanism of action of herbicides are associated to the modulation of enzymes that are essential for a plant’s growth and survival. In addition, among a vast number of herbicides, the class of Sulfonylureas and Imidazolinones are the most used to develop new tolerance crops, since it is already know how these molecules act in plants (Endo and Toki, 2013; Shoba et al., 2017). The development of herbicide tolerant plants have been achieved by introducing specific mutations (non-GM approach) in the target site of herbicide action (Green and Owen, 2011), since it has the advantage of easier registration/release for commercial cultivation as well as wider public acceptance (Fartyal et al., 2018). Among different kind of herbicides, Imidazolinones are probably the most widely targeted ones for developing herbicide tolerant crops through non-GM approach (Shoba et al., 2017).

Imidazolinones act by inhibiting acetolactate synthase (ALS) enzymes, which are involved in branched chain amino acid (Valine, Leucine and Isoleucine) biosynthesis (Piao et al., 2018). These molecules act blocking the substrate access channel, triggering the deficiency of the related amino acids (Garcia et al., 2017). The resulting reduction of protein synthesis slows down cell division and leads to reduced growth and finally death in plants (Yu and Powles, 2014; Piao et al., 2018). As animals lack ALS gene, these class of herbicides follow principle of selective toxicity, being used worldwide (Gutteridge et al., 2012; Piao et al., 2018). In addition, they are highly selective, very potent, and required only in small amounts (Endo et al., 2007). Since *Lactuca serriola* naturally evolved tolerance to an Imidazolinone, several ALS gene mutations have been identified blocking the binding of herbicides to ALS enzymes, contributing to herbicide tolerance in plants. ALS mutants have also been created artificially by site-directed mutagenesis *in vitro* (Chong and Choi, 2000), chemically induced mutagenesis (Koch et al., 2012), transcription activator-like effector nucleases mediated (TALEN) mutagenesis (Li et al., 2016), clustered regularly interspaced short palindromic repeats (CRISPR) mediated mutagenesis (Sun et al., 2016), and more recently by CRISPR-mediated homology-directed DNA repair (HDR) technology (Li et al., 2019). In rice, various mutant lines presenting specific amino acids changes in ALS are capable to tolerate different Imidazolinones, including a Glycine to Glutamate in codon 628 (G628E – Croughan, 1994), a Serine to Asparagine in codon 627 (S627N – Piao et al., 2018), and a double mutation of Tryptophan to Leucine in codon 548 (W548L)/Serine to Isoleucine in codon 627 (S627I) (Shimizu et al., 2002).

The combination of these mutant lines with the specific herbicides provides a reliable approach to eliminate weeds in the fields (Chauhan, 2013; Piao et al., 2018). However, the continuous overuse of a single herbicide multiple times in a growing season increases the potential risk of evolution of resistant weeds which has become a major concern in agriculture worldwide (Fartyal et al., 2018). For this reason, the discovery of new mutations in ALS to generate rice plants more tolerant to Imidazolinones than the available mutant rice lines is still a hot topic in plant-herbicide interaction field. Keeping that in mind, we carried out molecular docking experiments of Imidazolinone herbicides imazapic, imazapyr, imazaquin, and imazethapyr to evaluate the interaction of these molecules in the binding cavity of ALS (Os02g30630) from rice, being able to identify the most important amino acids responsible for the stability of these four herbicides. After introducing point mutations in these specific amino acid residues (one at a time) using Alanine scanning mutagenesis method and recalculating the effect in the affinity of herbicide-ALS interaction, we were able to propose novel mutation sites on the structure of ALS presenting a highest impact in the binding of Imidazolinones to ALS when compared to the already known amino acid mutations. To obtain a rice cultivar capable to tolerate the four Imidazolinone tested at the same time, we suggest six amino acid mutations at positions Val170, Phe180, Lys230, Arg351, Trp548, and Ser627 in the OsALS1.

## Materials and Methods

### Comparative Modeling

The homology modeling methodology, implemented in the MODELLER (Sali and Blundell, 1993) 9v19 program, was used in order to build the models for ALS1 from *Oryza sativa*. The structure of *Arabidopsis thaliana* ALS (PDB ID: 5K6T) was used as template. The protocol comprehends the generation of 10 models to built the structure of ALS1 from *O. sativa*. To select the best structure we submitted all models to the energy function DOPE (Shen and Sali, 2006). Furthermore, we also used the MOLPROBITY webserver (Chen et al., 2010) and PROCHECK (Laskowski et al., 1993) program to check the stereo chemical quality of the models.

### Molecular Docking Experiments

Protein-ligand interactions between the herbicides (Imazapic, Imazapyr, Imazaquin, and Imazethapyr) and OsALS1 structure were evaluated by molecular docking. The herbicide structures were obtained from the PubChem database (Kim et al., 2019). The hydrogens of the herbicide structures were added taking into account the pH 7.4, and a -1 charge were considered. All small molecules and receptor were prepared using AutoDockTools program, whereas the docking simulations were carried out with AutoDock4.2, allowing flexibility to the ligands (Morris et al., 1998). The estimated free energy of binding was provided by AutoDock4.2, and to define the best pose was used the most populated cluster. All docking experiments used the structure of *O. sativa* ALS. To ensure that all herbicides were properly docked, a 3D-grid with dimensions 40 × 40 × 40 with spacing of 0.375 Å was used to define the active site. The grid was defined in the herbicide binding site region, based on ALS structure of *A. thaliana*. Re-docking procedure was performed to evaluate whether the program could reproduce the ligand location found in the crystallographic structure (PDB ID 5K6T). Then, Lamarckian Genetic Algorithm was the search algorithm chosen with 50 runs, and the other parameters were set to their default values, except for number of evaluations and number of individuals in population, which were set to 2.500.000 and 300, respectively.

### Computational Alanine Scanning

Computational Alanine scanning approach is commonly used to identify residues important to the stability of protein-protein interfaces (Qiu et al., 2018). However, this methodology can also be applied to evaluate protein-ligand interactions, where a specific residue into the active site is mutated to Alanine, and the difference in binding free energies is computed. The mutations were performed to all residues that are five angstroms distant from the herbicides, and the estimated free energy of binding was computed by AutoDock4.2. The corresponding ΔΔG values were obtained by subtracting the mutant free energies of binding from the wild type. All evaluations were performed by the ABS-Scan program (Anand et al., 2014).

### Genomic DNA Extraction and OsALS1 Sequencing

Genomic DNA was extracted from rice leaves of two Imidazoline-susceptible (IRGA 424 and IRGA 417) and two Imidazolinone-tolerant (IRGA 424-RI and Puitá INTA-CL) cultivars using the PureLink^TM^ Genomic Plant DNA Purification Kit (Thermo Fisher Scientific). Both Imidazolinone-tolerant cultivars are essentially derived from the tested Imidazolinone-susceptible ones. Sequences of wild type ALS1 gene and protein (LOC_Os02g30630) were obtained from the Rice Genome Annotation Project^1^. To amplify 2,183 bp of *OsALS1* sequences we used the primers ALS1-F (CACACTCTCCACCCCTCTCT) and ALS1-R (AGGATTACCATGCCAAGCAC). Reaction settings were composed of an initial denaturation step of 10 min at 94°C, followed by 40 cycles of 45 s at 94°C, 45 s at 60°C, and 2 min at 72°C, followed by a final elongation step of 10 min at 72°C. PCRs were carried out in 25 μl final volume composed of 2.5 μl of 10× PCR buffer, 1.25 μl of 50 mM MgCl_2_, 1 μl of 10 mM dNTPs, 1 μl dimethyl sulfoxide (DMSO), 1 μl of each primer (10 μM), 2.5 U of Platinum Taq DNA Polymerase (5 U/μl, Invitrogen, Carlsbad, CA, United States), and 2 ng of genomic DNA. Amplicons were purified with Illustra GFX PCR DNA and Gel Band Purification Kit (GE Healthcare) and sequenced in the ACTGene Laboratory^2^ with an automatic sequencer Applied Biosystems 3500 Genetic Analyzer (Thermo Fisher Scientific), using both amplification primers (ALS1-F and ALS1-R) and two additional internal primers (ALS1-seqF: GGTCATCACCAACCACCTCT; ALS1-seqR: CAGTAGCGATGATTGCCTCA).

## Results and Discussion

### OsALS1 Structure and Active Site (Interaction of Imidazolinones With OsALS1)

**FIGURE 1:**
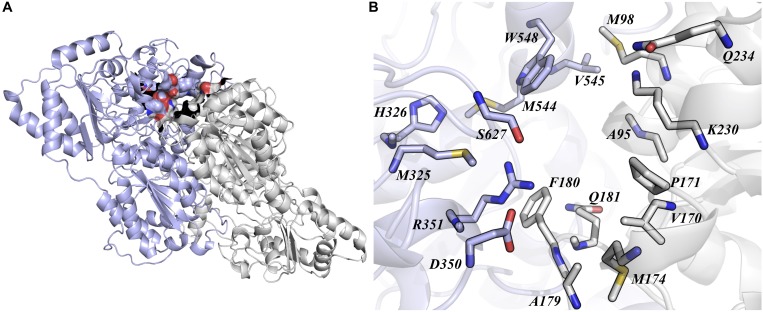
Quaternary packing of OsALS1 structure. **(A)** The active site of OsALS1 is located at the interface of the dimer. The active site is represented as surface. **(B)** All residues up to 5 Å of the ligand are represented as sticks and colored as CPK. The overall structure is represented as cartoon, and subunit **(A,B)** are colored in light blue and gray, respectively. Figure was generated using PyMOL.

Acetolactate synthase is the first enzyme in the background branched-chain amino acids (BCAA) pathway in plants and many microorganisms (Dezfulian et al., 2017). This enzyme is biologically active as a dimer and its active site is located at the interface of the subunits. The formation of the quaternary structure is dependent of the FAD coenzyme association, which is also necessary for the binding of α-ketoacid substrates and inhibitors (Ott et al., 1996). The primary OsALS1 sequence consist of 644 amino acid residues, and its tertiary structure is composed of 31.52% α-helices, 22.52% extended strands and 9.94% β-turn (Yaqoob et al., 2016). Our OsALS1 model was built using the structure of *A. thaliana* ALS (PDB ID: 5K6T) (Garcia et al., 2017) as template and follow the same fold described by Yaqoob et al. (2016; Figure 1A). In order to evaluate the affinity of different Imidazolinone herbicides (Imazapic, Imazapyr, Imazaquin, and Imazethapyr), we carried out molecular docking experiments. Garcia et al. (2017) described that Imidazolinone herbicides binds to ALS in the channel that leads to the active site. Therefore, we first determined a docking protocol to assure that a ligand conformation close to the crystallographic structure could be obtained. According to our results, Imazapic, Imazapyr, Imazaquin, and Imazethapyr compounds show similar binding modes, where the imidazole moiety is interacting to residues located close to the surface of the protein, whereas the same moiety of the Imazapyr molecule is buried in the cavity. Despite the orientation differences of these compounds, the binding affinity predicted by AutoDock score function are within the standard deviation. According to our docking analysis, we identified 20 residues (G95, A96, M98, V170, P171, M174, A179, F180, Q181, K230, Q234, M325, H326, D350, R351, M544, V545, W548, S627, and G628) that are participating in the binding of these herbicides (Figure 1B). Some of these mutations (A96, P171, A179, W548, and S627) commonly occur conferring tolerance to ALS inhibitors (Tranel and Wright, 2002; Tan et al., 2005).

### Identification of Key Residues for OsALS1-Imidazolinones Interaction

In order to identify key residues responsible for the interaction of Imidazolinones into the OsALS1 binding site, we applied the computational Alanine scanning approach followed by an energy evaluation process. As described by our docking experiments, we identified 20 residues that are up to 5 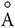 closed to the herbicide into the channel that leads to the active site. All these residues were mutated to Alanine, bringing a total of 20 mutant models of OsALS1. The binding affinities to all mutant models were calculated and compared to the wild type (Supplementary Tables S1–S4). Since all herbicides associate into the same binding cavity, the number of residues that interact are similar, ranging from 13 to 15. Imazapyr is the molecule that presents the highest number of interactions, while Imazaquin presents the lowest number. Also, Imazapyr compound have preference to interact with polar residues, since 10 of the 15 interactions are maintained by Aspartate, Arginine, Serine, Glutamine, Glutamate, and Lysine, whereas Imazapic, Imazaquin, and Imazethapyr do not show preferences to a specific type of amino acid, interacting with the same number of polar and non-polar residues. In addition, the conformation of Imazapyr into the binding site differs from the others, where the imidazole ring is buried in the cavity, while Imazapic, Imazaquin, and Imazethapyr have the same portion positioned at the entrance of the binding pocket. These different conformations could explain why the most important residues are not the same to all herbicides.

**FIGURE 2:**
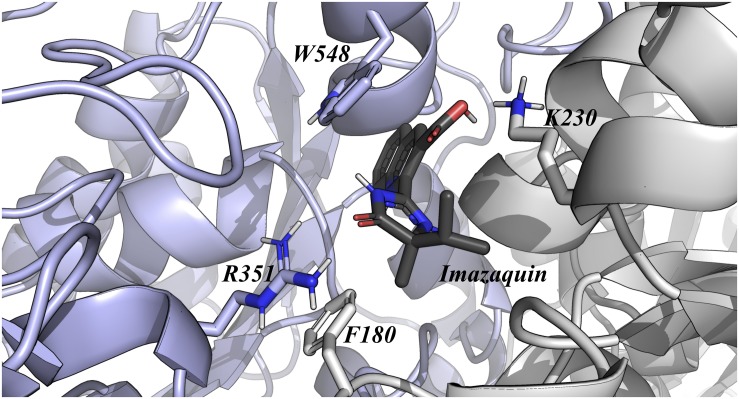
OsALS1 enzyme associated with Imazaquin. Only the OsALS1 binding site is shown. Imazapic, Imazaquin, and Imazethapyr show the same position at the active site. Imazaquin compound and the most important residues responsible for ligand recognition are shown in sticks. The protein residues are colored according to CPK, except the carbon that is presented in light purple. In addition, Imazaquin is also colored by CPK, except the carbon atom that is presented in dark gray. Figure was generated using PyMOL.

Analysis of Alanine scanning revealed that the main residues guiding the interaction of OsALS1 and Imazapic, Imazaquin, and Imazethapyr were Phe180, Lys230, Arg351, and Trp548 (Figure 2). Among all these point mutations, we were able to highlight two promising positions, which could be used to improve OsALS1 tolerance against Imidazolinones. With regards to Imazapic, Imazaquin, and Imazethapyr, the single mutation Lys230Ala decreases approximately 31, 31, and 30%, while the single mutation Trp548Ala decreases 24, 22, and 25% the affinity by the OsALS1, respectively. According to our docking experiments, the side chain of Trp548 is involved in π-stacking interactions, whereas the side chain of Lys230 is maintaining electrostatic interactions with these molecules. Based on the position of these mutation and its impact on the relative binding affinity, we suggest that Lys230Ala could be a more interesting mutation site when compared to Trp548Ala.

**FIGURE 3:**
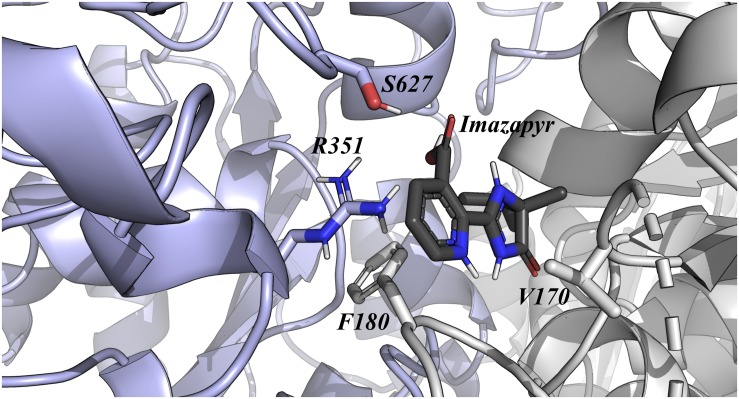
OsALS1 enzyme associated with Imazapyr. Only the OsALS1 binding site is shown. Imazapyr compound and the most important residues responsible for ligand recognition are shown in sticks. The protein residues are colored according to CPK, except the carbon that is presented in light purple. In addition, Imazapyr is also colored by CPK, except the carbon atom that is presented in dark gray. Figure was generated using PyMOL.

The relative binding affinity of Imazapyr was most affected by mutations at Val170, Phe180, Arg351, and Ser627, decreasing approximately 6, 7, 21, and 7%, respectively (Figure 3). In addition, it is interesting to observe that one of the most important residue identified by this approach (Ser627) has already been described as a critical amino acid for Imidazolinone tolerance. However, our result suggests that Arg351Ala mutation could enhance even more the tolerance of this enzyme to Imazapyr. Together, these analyses of single point mutations reveal that the tolerance of OsALS1 against Imidazolinones compounds could be improved by two new mutations sites at the positions Lys230 and Arg351.

### Effect of Multiple Mutations on the Putative Tolerance of OsALS1 to Imidazolinones

After identifying key residues for OsALS1-Imidazolinones interaction, we decided to check the effect of multiple mutations on the putative tolerance of OsALS1 to different Imidazolinones. As commented before, Imazapic, Imazaquin, and Imazethapyr presented a similar pattern of interaction with OsALS1 enzyme, being Lys230 the single mutation that most affects OsALS1 interaction with the three Imidazolinones (Table 1), while the other three single mutations (Phe180, Arg351, and Trp548) affected the estimated free energy of binding in a lesser extent. When two, three, and finally all the four mutations were analyzed, we detected a constant decrease in the free energy of binding. The most effective double and triple mutations were Lys230/Trp548 and Lys230/Arg351/Trp548, respectively. The quadruple mutation (Phe180/Lys230/Arg351/Trp548) was even more effective, and the estimated free energy of binding was decreased 67% for Imazapic, 62% for Imazaquin, and 64% for Imazethapyr, when compared to the wild type enzyme (Table 1).

**TABLE 1:** Estimated free energy of binding for single and multiple mutations identified by Alanine scanning approach on OsALS1 associated with Imazapic, Imazaquin, and Imazethapyr.

Mutations	Estimated free energy of binding (kcal/mol)		
	Imazapic	Imazaquin	Imazethapyr
Wild type	−7.55	−8.25	−7.64
Phe180Ala	−7.07	−7.80	−7.20
**Lys230Ala**	**−5.20**	**−5.81**	**−5.25**
Arg351Ala	−6.74	−7.73	−7.09
Trp548Ala	−5.89	−6.23	−5.84
Phe180Ala_Lys230Ala	−4.68	−5.56	−4.97
Phe180Ala_Arg351Ala	−5.66	−6.87	−6.09
Phe180Ala_Trp548Ala	−4.93	−5.63	−5.10
Lys230Ala_Arg351Ala	−4.35	−5.36	−4.71
**Lys230Ala_Trp548Ala**	**−3.63**	**−4.12**	**−3.72**
Arg351Ala_Trp548Ala	−4.60	−5.42	−4.83
Phe180Ala_Lys230Ala_Trp548Ala	−3.22	−3.73	−3.36
Phe180Ala_Arg351Ala_Trp548Ala	−4.20	−5.04	−4.48
Phe180Ala_Lys230Ala_Arg351Ala	−3.95	−4.98	−4.35
**Lys230Ala_Arg351Ala_Trp548Ala**	**−2.90**	**−3.52**	**−3.09**
Phe180Ala_Lys230Ala_Arg351Ala_ Trp548Ala	−2.49	−3.14	−2.73

*The most effective single, double, and triple mutations were highlighted in bold.*

On the other hand, Imazapyr presents an interaction mode different from the other herbicides, being Arg351 the single mutation that most affects OsALS-Imazapyr interaction (Table 2). As well as with Imazapic, Imazaquin, and Imazethapyr, the other three single mutations (Val170, Phe180, and Ser627) were less effective, showing higher levels of estimated free energy of binding. We also detected a continuous decrease in the estimated free energy of binding according to the number of mutations in OsALS1 enzyme. The most effective double and triple mutations for Imazapyr were Arg351/Ser627 and Phe180/Arg351/Ser627, respectively, while the quadruple mutation Val170/Phe180/Arg351/Ser627 decreased 44% the estimated free energy of binding when compared to the wild type enzyme (Table 2).

**TABLE 2:** Estimated free energy of binding for single and multiple mutations identified by Alanine scanning approach on OsALS1 associated with Imazapyr.

Mutations	Estimated free energy of binding (kcal/mol)
	Imazapyr
Wild type	−7.30
Val170Ala	−7.15
Phe180Ala	−6.78
**Arg351Ala**	**−5.77**
Ser627Ala	−6.77
Val170Ala_ Phe180Ala	−6.78
Val170Ala_Arg351Ala	−5.91
Val170Ala_Ser627Ala	−6.46
Phe180Ala_ Arg351Ala	−5.80
Phe180Ala_Ser627Ala	−6.40
**Arg351Ala_Ser627Ala**	**−5.51**
Val170Ala_Phe180Ala_Arg351Ala	−5.53
Val170Ala_Phe180Ala_Ser627Ala	−6.08
Val170Ala_Arg351Ala_Ser627Ala	−5.14
**Phe180Ala_Arg351Ala_Ser627Ala**	**−5.14**
Val170Ala_Phe180Ala_Arg351Ala_Ser627Ala	−4.82

*The most effective single, double, and triple mutations were highlighted in bold.*

Most of the Imidazolinone-tolerant rice cultivars are currently developed from either one or a combination of Ala179, Trp548, and Ser627 mutations (Tan et al., 2005; Shoba et al., 2017; Piao et al., 2018; Li et al., 2019), with the combination Trp548 and Ser627 being the most recurrent. It is interesting to note that the most effective double and triple mutations detected in our analysis to the four Imidazolinones encompass only one of the classical mutations (Trp548 for Imazapic, Imazaquin, and Imazethapyr; Ser627 for Imazapyr), while the other putative mutations are novel and could be even more effective (Lys230 for Imazapic, Imazaquin, and Imazethapyr; Arg351 for Imazapyr).

### Validation of Bioinformatics Data Using Traditional Rice Cultivars

In order to validate the data obtained by molecular docking of OsALS-Imidazolinones, we checked the sequence of *OsALS1* in four Brazilian rice cultivars that present contrasting response to Imidazolinones: IRGA 424 and IRGA 417 (both susceptible); IRGA 424-RI (tolerant); and Puitá INTA-CL (highly tolerant). Both Imidazolinone-tolerant cultivars (IRGA 424-RI and Puitá INTA-CL) were obtained by conventional mutation breeding/selection of IRGA 424 and IRGA 417, respectively. As seen in Table 3, both imidazoline-susceptible cultivars present the same amino acid residues of the wild type (Val170, Phe180, Lys230, Arg351, Trp548, and Ser627). As expected, estimated free energies of binding were equal to the wild type, considering all the Imidazolinones.

**TABLE 3:** Estimated free energy of binding for the OsALS1 mutants proposed by computational analyses and four Brazilian cultivars and their interactions with Imazapic, Imazaquin, Imazethapyr, and Imazapyr.

		Amino acid position						Estimated free energy of binding (kcal/mol)			
	Imidazoline response	170	180	230	351	548	627	Imazapic	Imazaquin	Imazethapyr	Imazapyr
**Wild type**	**Susceptible**	Val	Phe	Lys	Arg	Trp	Ser	−7.55	−8.25	−7.64	−7.30
**IRGA 424**	**Susceptible**	Val*	Phe*	Lys*	Arg*	Trp*	Ser*	−7.55	−8.25	−7.64	−7.30
**IRGA 424−RI**	**Tolerant**	Thr	Arg	Ala	Arg*	Trp*	Ser*	−5.51	−6.44	−5.81	−7.17
**IRGA 417**	**Susceptible**	Val*	Phe*	Lys*	Arg*	Trp*	Ser*	−7.55	−8.25	−7.64	−7.30
**Puitá INTA−CL**	**Highly tolerant**	Ala	Ser	−**	Arg*	Trp*	Ser*	−2.82	−2.96	−3.44	−5.10
**Deletion_1**	**Highly tolerant (?)**	Ala	Ala	−**	Ala	Ala	Ala	−2.21	−2.75	−2.05	−4.22
**Deletion_2**	**Highly tolerant (?)**	Ala	Ala	Ala	−**	Ala	Ala	−1.94	−2,43	−1.19	−4.40
**Recommendation**	**Highly tolerant (?)**	Ala	Ala	Ala	Ala	Ala	Ala	−2.08	−2.70	−2.25	−4.51

*No substitution when compared with the wild type sequence. **Deletion.

The tolerant IRGA 424-RI cultivar presented three mutations (Val170Thr, Phe180Arg, and Lys230Ala) when compared to the wild type sequence, which moderately impacted the estimated free energy of binding of Imazapic, Imazaquin, and Imazethapyr, but not of Imazapyr (Table 3). Such difference is probably explained by the fact that Lys230 (mutated in IRGA 424-RI) is the single mutation that most affects OsALS1 interaction with Imazapic, Imazaquin, and Imazethapyr, while Arg351 (not mutated in IRGA 424-RI) is the single mutation that most affects OsALS-Imazapyr interaction. The highly tolerant Puitá INTA-CL cultivar also presented three mutations (Val170Ala, Phe180Ser, and Lys230deletion) when compared to the wild type sequence, which strongly impacted the interaction of OsALS1 with Imazapic, Imazaquin, and Imazethapyr (as expected due to the lack of Lys230), and moderately impacted OsALS-Imazapyr interaction (even without the mutation in position Arg351) (Table 3). Therefore, it seems that amino acid deletion at position 230 is more impacting than amino acid substitution. It is interesting to highlight that none of the Imidazolinone-tolerant cultivars present mutations in positions Arg351, Trp548, and Ser627, suggesting that these cultivars could be even more tolerant if such mutations were introduced.

Considering that usually the Imidazolinone-based herbicides are composed by a mixture of different compounds, it would be interesting to test the effect of all six promising mutations identified in our work as more important for the binding of all Imidazolinones. When the six amino acids are mutated to Alanine, we obtain very low values of free energy of binding, considering all tested herbicides (Table 3), suggesting that such approach could be used to generate rice cultivars with high levels of tolerance to the four imidazolines concomitantly. Stimulated by the deletion found on the tolerant Puitá INTA-CL cultivar, we tested other two constructions: Alanine substitution in five amino acid positions, along with deletion of the two most important amino acids for OsALS-Imidazolinones interaction, Lys230 and Arg351. The estimated free energies of binding were very close to the six Alanine substitutions approach, and therefore we only suggest the deletion of Lys230, or preferably Arg351 (coupled with substitution of the other five amino acids to Alanine), in order to inhibit OsALS-Imazethapyr interaction.

### Estimating the Effects of the Proposed Mutations on the Stability of Acetolactate Synthase

In order to evaluate the effects of the proposed mutations (Val170Ala, Phe180Ala, Lys230Ala, Arg351Ala, Trp548Ala, and Ser627Ala) on the stability of ALS structure, we carried out a computational analysis using the web server Site Directed Mutator (SDM) (Worth et al., 2011). A detail description of the parameters of the SDM web server is given elsewhere (Worth et al., 2011). According to SDM server, mutations can be classified as stabilizing, when the changes in Gibbs free energy (ΔΔG) is ≥ 0.0 kcal/mol, or destabilizing when ΔΔG is <0. The analysis shown that five mutations have no deleterious effect on the protein stability, whereas Val170Ala presented a ΔΔG of -0.53 indicating a destabilizing mutation (Supplementary Table S5). However, the impact of this mutation seems to have no negative effect on the protein function, since Puitá INTA-CL cultivar carries this mutation (Table 3). It is important to note that all proposed mutations are not located into the catalytic site, since this class of herbicides have a different binding pocket located in the channel that leads to the active site.

## Conclusion

Altogether, the data presented here indicate that Imidazolinone tolerance in rice can be increased by inserting mutations in specific (and previously unknown) amino acid residues of OsALS1 enzyme. Such bioinformatics results (corroborated by sequencing of *OsALS1* gene in different tolerant and susceptible rice cultivars) could be used rationally to generate tolerance to a specific Imidazolinone or even to the four Imidazolinones at the same time. The identification of new promising amino acid residues important to OsALS-Imidazolinone interaction is of particular importance if we take into account the continuous overuse of a single herbicide multiple times, and the increasing potential risk for weeds to develop tolerance.

## Data Availability Statement

The raw data supporting the conclusions of this article will be made available by the authors, without undue reservation, to any qualified researcher.

## Author Contributions

GB, ML, RS, and LT conceived and designed research. GB, TL, and LT conducted experiments. ML contributed with analytical tools. GB and LT analyzed the data. GB, RS, and LT wrote the manuscript. All authors read and approved the manuscript.

## Conflict of Interest

Patent submitted to INPI (Instituto Nacional da Propriedade Intelectual) in 2018 under registration number BR1020180740490, entitled “Linhagem de Arroz Geneticamente Modificado, Seu Processo de Produção e Método de Controle de Plantas Daninhas”. The authors declare that the research was conducted in the absence of any commercial or financial relationships that could be construed as a potential conflict of interest.

## Footnotes

1 http://rice.plantbiology.msu.edu/

2 https://actgene.com.br/
