# Supplementary material for: Push It to the Limit: Identification of Novel Amino Acid Changes on the Acetolactate Synthase Enzyme of Rice That Putatively Confer High Level of Tolerance to Different Imidazolinones
Source: Front Bioeng Biotechnol. 2020 Feb 14;8:73. doi: 10.3389/fbioe.2020.00073 (PMC7033567; doi:10.3389/fbioe.2020.00073)
Supplement: Supplementary file 1 [file Table_1.docx]

**Supplementary material**

**Supplementary Table 1**. Estimated free energy of binding for single mutations identified by Alanine scanning approach on OsALS1 associated with Imazapic. The most effective mutations were highlighted in bold.

| ***Mutations*** | ***Estimated Free Energy of Binding (kcal/mol)*** |
| --- | --- |
| Wild type | -7.56 |
| Ala96Ala | -7.55 |
| **Met98Ala** | **-7.25** |
| Ser142Ala | -7.56 |
| Val170Ala | -7.45 |
| Ala179Ala | -7.55 |
| **Phe180Ala** | **-7.07** |
| Gln181Ala | -7.46 |
| **Lys230Ala** | **-5.20** |
| Asp350Ala | -7.65 |
| **Arg351Ala** | **-6.75** |
| Met544Ala | -7.54 |
| Val545Ala | -7.46 |
| **Trp548Ala** | **-5.90** |
| Tyr553Ala | -7.52 |
| Ser627Ala | -7.39 |
|  |  |

**Supplementary Table 2**. Estimated free energy of binding for single mutations identified by Alanine scanning approach on OsALS1 associated with Imazaquin. The most effective mutations were highlighted in bold.

| ***Mutations*** | ***Estimated Free Energy of Binding (kcal/mol)*** |
| --- | --- |
| Wild type | -8.26 |
| Ala96Ala | -8.26 |
| **Met98Ala** | **-7.96** |
| Gln169Ala | -8.27 |
| Val170Ala | -8.18 |
| **Phe180Ala** | **-7.81** |
| **Lys230Ala** | **-5.82** |
| Aps350Ala | -8.36 |
| **Arg351Ala** | **-7.74** |
| Met544Ala | -8.11 |
| Val545Ala | -8.08 |
| **Trp548Ala** | **-6.23** |
| Tyr553Ala | -8.24 |
| Ser627Ala | -8.22 |
|  |  |

**Supplementary Table 3**. Estimated free energy of binding for single mutations identified by Alanine scanning approach on OsALS1 associated with Imazapyr. The most effective mutations were highlighted in bold.

| ***Mutations*** | ***Estimated Free Energy of Binding (kcal/mol)*** |
| --- | --- |
| Wild type | -7.31 |
| Ala96Ala | -7.31 |
| Thr141Ala | -7.30 |
| Ser142Ala | -6.68 |
| Gln169Ala | -7.33 |
| **Val170Ala** | **-6.93** |
| Arg173Ala | -7.18 |
| Met174Ala | -7.09 |
| Ala179Ala | -7.25 |
| **Phe180Ala** | **-6.79** |
| **Gln181Ala** | **-7.06** |
| Glu182Ala | -7.39 |
| Lys230Ala | -7.06 |
| **Arg351Ala** | **-5.78** |
| Trp548Ala | -7.26 |
| **Ser627Ala** | **-6.57** |
|  |  |

**Supplementary Table 4**. Estimated free energy of binding for single mutations identified by Alanine scanning approach on OsALS1 associated with Imazethapyr. The most effective mutations were highlighted in bold.

| ***Mutations*** | ***Estimated Free Energy of Binding (kcal/mol)*** |
| --- | --- |
| Wild type | -7.65 |
| Ala96Ala | -7.64 |
| **Met98Ala** | **-7.33** |
| Ser142Ala | -7.65 |
| Val170Ala | -7.53 |
| Ala179Ala | -7.64 |
| **Phe180Ala** | **-7.20** |
| Gln181Ala | -7.57 |
| **Lys230Ala** | **-5.26** |
| Asp350Ala | -7.74 |
| **Arg351Ala** | **-7.10** |
| Met544Ala | -7.59 |
| Val545Ala | -7.49 |
| **Trp548Ala** | **-5.85** |
| Tyr553Ala | -7.61 |
| Ser627Ala | -7.61 |
|  |  |

**Supplementary Table 5**. Computational site-mutation experiments on the proposed positions Val170, Phe180, Lys230, Arg351, Trp548, and Ser627 of Acetolactacte synthase enzyme. All analyses were carried out using SDM web server.

| Structure | Wild type | Mutation | **ΔΔG (kcal/mol)** | Effect |
| --- | --- | --- | --- | --- |
| OsALS1 | Val170 | Alanine | -0.53 | Reduced stability |
|  | Phe180 |  | 0.78 | Increased stability |
|  | Lys230 |  | 0.47 | Increased stability |
|  | Arg351 |  | 0.41 | Increased stability |
|  | Trp548 |  | 0.47 | Increased stability |
|  | Ser627 |  | 0.62 | Increased stability |
